# Eyes, the window on psychosis?

**DOI:** 10.1192/bjo.2022.16

**Published:** 2022-02-10

**Authors:** Natalie Shoham, Claudia Cooper

**Affiliations:** Division of Psychiatry, University College London (UCL), UK; and Camden and Islington NHS Foundation Trust, St Pancras Hospital, London, UK; Division of Psychiatry, University College London (UCL), UK; and Camden and Islington NHS Foundation Trust, St Pancras Hospital, London, UK

**Keywords:** Visual impairment, schizophrenia, psychosis, congenital blindness, visual acuity

## Abstract

Much has been written on the theory that congenital blindness might protect against schizophrenia, but proof remains elusive. It has been suggested that visual ability might be associated with schizophrenia in a bell-shaped distribution, with both lifelong absent and perfect vision being protective. Alternatively, ocular aberrations and schizophrenia may share an aetiology. Any neuronal pathology implicated in schizophrenia could affect the retina, since it is an embryological extension of the brain. The retina is more amenable to direct imaging than other parts of the central nervous system and may give unique insights into schizophrenia-associated neuropathology. It is also possible that psychosis causes visual impairment: people with psychotic illnesses are probably not accessing optical care optimally and have higher levels of risk factors for visual loss.

Cross-sectionally, there is an association between visual impairment and psychosis, in both younger and older adults.^1^ Longitudinal evidence, however, paints a mixed picture. A study of over 1 million male military conscripts in Sweden found that poorer visual acuity in adolescence increased the likelihood of a subsequent diagnosis of psychotic illness.^2^ The association remained when siblings were used as controls to account for both genetic and environmental confounders.^2^ A very similar study in Israel found the exact reverse: refractive error in adolescence was negatively associated with schizophrenia in the future.^3^ The Swedish study suggested that correcting vision using glasses or similar aids does not remove the association, although it may attenuate it.^2^ The reversed findings between this and the Israeli study are difficult to explain. Of note, however, is that the Israeli study does not specify duration of follow-up or which cut-off was used to define refractive error, and reported an unusually low prevalence of refractive error, suggesting that the cut-off was stringent.^3^ Myopia is positively associated with educational level, but both studies adjusted for possible negative confounding by IQ or socioeconomic status.

## The ‘protection against schizophrenia’ model

Since 1950, multiple authors have commented on a supposed absence of reported cases of a congenitally blind person developing schizophrenia.^4^ At the most extreme, some have claimed that there are ‘no blind schizophrenics’.^5^ The ‘protection against schizophrenia’ model suggests that there is a roughly bell-shaped relationship between level of vision and psychosis, with both perfect and congenitally absent vision being protective.^6^ This is not to suggest that vision itself is a risk factor; rather that aberrant visual input is, and absent vision prevents this.^4^ Silverstein and colleagues report in detail the potential mechanisms through which congenital blindness could be protective.^4^ For example, congenitally blind individuals have superior auditory processing abilities to sighted individuals, and better working memory to enable them to create mental representations of objects through sequential touch. These are the reverse of typical cognitive deficits seen in schizophrenia, so a buffering effect might be created whereby congenitally blind individuals are less susceptible.^4^ In individuals with severe vision *loss*, spontaneous activity of the visual cortex appears able to drive visual hallucinations.^7^ This experience has not been reported in people with congenital blindness even when hallucinogenic drugs are taken, and it is posited that lack of stored visual representations or recruitment of the visual cortex for processing of alternative sensory information may prevent it.^7^ Further, dark-rearing of animals has been shown to alter *N*-methyl-d-aspartate (NMDA) receptor structure and function.^5^ Sanders and colleagues present the hypothesis that NMDA receptor adaptations in the occipital cortex may extend to the anterior cingulate cortex, which is a functionally connected region and has a role in cognition and schizophrenia, leading to the protective effect.^5^ These theories are not specific to any one cause of congenital blindness.

Sceptics of the theory that congenital blindness protects against schizophrenia argue that to statistically prove a negative association between these two conditions, databases of many millions of participants would be required; and these have not so far been used to test the hypothesis.^8^ Nevertheless, shared risk factors for the two conditions, such as birth trauma and chromosomal abnormalities, would seem to make the lack of case reports conspicuous.

A 2014 revisitation of the literature found possible reports of psychosis in congenitally blind individuals, challenging earlier assertions of their absence.^9^ However, most of these examples did not appear to describe psychotic illness by modern diagnostic criteria, with one clearly describing autism instead.^4^ All individuals described had peripheral blindness (originating from the eye) as opposed to cortical blindness, leading the authors to conclude that it might be specifically congenital cortical blindness that is protective.^9^

## Impaired visual processing and shared brain–eye neuropathology

Visual processing is much broader than visual acuity. It includes lower-level functions, some of which occur at eye level, such as visual acuity; medium-level functions, such as visual perceptual organisation or ‘putting together’ of visual components; and higher-level functions, such as using existing knowledge to adapt visual perceptions.^10^ An association between schizophrenia and visual processing impairment more broadly is well established.^10^ In fact, a US trial of visual cognitive remediation therapy in schizophrenia is currently underway, to determine whether improving visual processing leads to gains in cognition and functioning more generally.^10^ Studies investigating visual processing impairment tend to assume that any impairment occurs at the level of the cortex, but as the lower-level eyesight functions are essential to this ability, a contribution from visual acuity impairment is difficult to discount.

The association between visual impairment and psychosis might also be explained, at least in part, by shared neuropathology between the brain and the eye. The retina is formed from the same embryonic tissue as the brain and central nervous system. It is therefore potentially affected by alterations in expressions of neurotransmitters, neuronal loss or neurodevelopmental abnormalities in the same way. Retinal imaging studies using structural (optical coherence tomography, OCT) imaging show macular thinning in people with schizophrenia, as well as alterations in retinal vasculature.^11^ Studies using functional (electroretinogram, ERG) imaging in schizophrenia consistently show reduced b-wave amplitude, with the b-wave primarily reflecting depolarisation of retinal bipolar and amacrine cells.^11^ To what extent retinal alterations translate to reduced vision is not entirely clear.^11^ Speculation that retinal imaging might one day have prognostic or diagnostic utility in schizophrenia has not yet been realised. The information obtainable through these techniques is limited; they cannot show neuronal structures beyond the eye, or synaptic functioning. Nevertheless, retinal imaging has shown promise as a tool for prevention and early diagnosis in Alzheimer's disease, and retinal alterations might be a marker of neurodegeneration or neural damage in multiple sclerosis, Huntington's disease, Parkinson's disease and traumatic brain injury, with early work also showing changes in bipolar disorder and autism.^11^ Hébert and colleagues have found retinal functional alterations in non-affected offspring of people with schizophrenia and bipolar disorder, suggesting that these may be trait characteristics;^12^ whereas Balogh and colleagues found that the alterations were state-related and occurred only in the acute phase of the illness.^13^

## Psychosis as a risk factor for visual impairment

We have so far considered visual impairment as a possible risk factor for, or marker of, schizophrenia. The converse relationship, whereby psychotic illnesses lead to poorer vision, has been less well-studied. Schizophrenia could very plausibly lead to visual impairment. Potential mechanisms include side-effects of antipsychotic medications, which can cause blurred vision through anticholinergic side-effects, as well as increasing the risk of glaucoma and cataracts.^14^ Older medications, such as thioridazine, can directly lead to damaging retinal deposits.^14^ Common comorbidities of psychotic illnesses, such as diabetes and hypertension, also increase the risk of eyesight damage. Psychotic illness may make it harder for people to arrange eye tests and other optical care, since this typically relies on individual initiative and obtaining funds. In fact, evidence suggests that a very high proportion of in-patients with psychotic illnesses both have visual acuity impairments and have not attended for eyesight testing for several years.^15^ In the USA, eyesight testing is recommended every 1–2 years as part of the routine physical health check for people with psychotic illnesses. By contrast in the UK, routine screening in not recommended in guidelines and not typically carried out in clinical practice.

## Conclusions

In this brief review, we have discussed three possible mechanisms which could explain the cross-sectional association between visual impairment and psychosis. Some researchers, notably Silverstein and colleagues, hope that improving visual processing has potential to improve overall functioning in people with schizophrenia. Theory regarding a protective effect of congenital blindness and causative effect of later-life visual impairment is described elegantly, but observational evidence is not yet conclusive. While we aim to further our understanding of the mechanisms by which visual impairment and psychosis associate, we should not forget the basics: how often do we ask our patients with schizophrenia when they last had their eyes tested?

## Data Availability

Data availability is not applicable to this article as no new data were created or analysed in its preparation.
